# Microstructure and Wear Behavior of In-Situ NbC Reinforced Composite Coatings

**DOI:** 10.3390/ma13163459

**Published:** 2020-08-05

**Authors:** Baoming Shi, Shiming Huang, Ping Zhu, Changen Xu, Tengfei Zhang

**Affiliations:** School of Materials Science and Engineering, Dalian Jiaotong University, Dalian 116028, China; S1455291509@163.com (B.S.); zhuping@djtu.edu.cn (P.Z.); xuchangen2003@sina.com (C.X.); koalahsm@sina.com (T.Z.)

**Keywords:** in-situ, composite coating, microstructure, NbC, wear resistance

## Abstract

In the present study, plasma spray welding was used to prepare an in-situ niobium carbide (NbC) reinforced Ni-based composite coating on the low carbon steel, and the phase composition and the microstructure of the composite coatings were studied. The wear resistance and the wear mechanism of the composite coatings were also researched by the wear tests. The results showed that the main phases of the composite coating were NbC, γ-Ni, Cr_23_C_6_, Ni_3_Si, CrB, Cr_5_B_3_, Cr_7_C_3_ and FeNi_3_. A number of fine in-situ NbC particles and numerous chromium carbide particles were distributed in the γ-Ni matrix. The increase in the mass fraction of Nb and NiCr-Cr_3_C_2_ could lead to the increase in NbC particles in the composite coatings. Due to the high hardness of NbC and chromium carbides, the micro-hardness and the wear resistance of the composite coatings were advanced. The composite coating with the powder mixtures of 20% (Nb + NiCr-Cr_3_C_2_) and 80% NiCrBSi had the highest micro-hardness and the best wear resistance in this study. The average micro-hardness reached the maximum value 1025HV_0.5_. The volume loss was 39.2 mm^3^, which was merely 37% of that of the NiCrBSi coating and 6% of that of the substrate under the identical conditions.

## 1. Introduction

With the rapid development of modern industry, machines have been charged with more and more duties. Hence, machine components and parts are subjected to severe wear conditions and need surfaces with prominent wear resistance. Several surface modification techniques, such as chemical vapor deposition [1,2], thermal spray [3,4,5], and laser cladding [6,7,8,9,10], have been applied to prepare strengthened coatings in order to enhance the wear resistance. Among the different surface modification techniques, plasma spray welding is a new fabrication technique with many advantages such as low operational cost, flexibility, high deposition rate, metallurgical bonding in the coating/substrate, and reduced probability of the existence of pores in the coatings. Therefore, there is widespread use in surface strengthening of machine components to resist the aggression environment.

There are many kinds of wear-resistant materials, including the particle reinforced metal matrix composite coatings studied in this study whose basic structure includes a relatively soft matrix and many hard particles. The hard particles in these coatings act as a wear resistant barrier to abrasive grit [11]. This structure can be achieved by two common methods: the hard particles can be synthesized separately prior to the fabrication process and then introduced to the metal matrix (ex-situ); the hard particles can be synthesized and separated out from molten metal during the composite fabrication (in-situ). The particle reinforcements are limited by the initial particle size in ex-situ particle reinforced composite coatings [12]. Besides, the properties of composite coatings are decreased on account of the poor wettability and the interfacial reactions between the reinforcements and the metal matrix [13]. In the molten pool, metallurgical reactions occur by means of the contacting of the raw materials, and the reinforced particles are in-situ synthesized during the fabrication process. In-situ particle reinforced composite coatings have many advantages over ex-situ particle reinforced coatings, such as uniform dispersion of reinforcements in the metal matrix, finer grain size, and that they are more cost-effective [14,15,16,17]. It is worth mentioning that the interface between the reinforcements and the metal matrix is stable and defect-free resulting in strong interfacial bonding.

Ceramic materials such as borides, nitrides and carbides are often used as reinforcements to enhance the performance of the coatings [18,19,20,21]. Therefore, ceramic particle reinforced metal matrix composite coatings with both metal properties (good toughness and ductility) and ceramic properties (high chemical stability and strength) could help in enhancing the wear resistance of the material surface [22,23]. With respect to the type of particle reinforcements, niobium carbide (NbC) can be considered as an attractive reinforcement for particle reinforced metal matrix composite coatings on account of its high hardness, high elastic modulus, good oxidation resistance and high wear resistance [24,25,26,27]. However, it is difficult to obtain a uniform microstructure for the coatings prepared by laser cladding with the preplaced powders due to the binding materials which will cause the burning loss of the powders [28]. By contrast, the coatings prepared by plasma spray welding have a good surface quality and large areas of uniform microstructure because the coaxial powder feeding is simultaneous with plasma spray welding and the powders are fused sufficiently in the molten pool. So far, the report on the preparation of in-situ NbC particles by plasma spray welding is relatively limited.

In the present work, in order to obtain in-situ NbC reinforced Ni-based composite coatings, plasma spray welding was put to use with the powder mixtures of NiCrBSi/Nb/NiCr-Cr_3_C_2_. NiCrBSi powders have an approximate coefficient of thermal expansion with the steel substrate and have a good wettability to the substrate. NiCr-Cr_3_C_2_ powders could not only offer the C source for the reaction of NbC, but they also promote the precipitation of chromium carbide. Authors aimed to investigate the composite coatings in terms of their microstructural characterization and wear behavior. Moreover, the wear mechanism of the composite coatings was also discussed.

## 2. Materials and Methods

**Figure 1:**
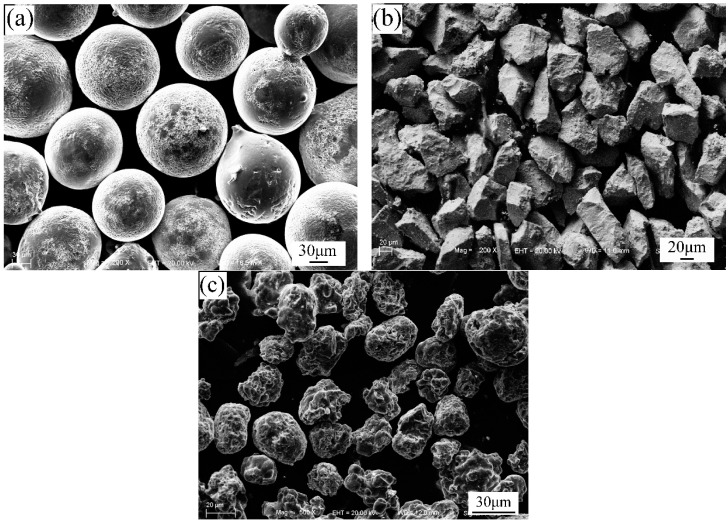
Morphologies of the raw materials in SEM (**a**) NiCrBSi, (**b**) Nb, and (**c**) NiCr-Cr_3_C_2_.

Commercial Q235 low carbon steel (200 mm × 100 mm × 10 mm in size) with nominal compositions (wt.%) of C: 0.12–0.20, Mn: 0.30–0.70, Si: ≤0.30, S: ≤0.045, P: ≤0.045 and Fe in balance were selected as the substrate material. Before welding, the surface of the steel substrate should be grinded, and then wiped with acetone to remove the oil. The spray welding powders are selected from commercially available NiCrBSi powders, Nb powders (with a purity of 99.9%) and NiCr-Cr_3_C_2_ powders, of which the purity of Nb powder is 99.9%. Figure 1 is the morphologies of the raw materials in SEM, and the chemical compositions are given in Table 1. It is clear that NiCrBSi, Nb and NiCr-Cr_3_C_2_ particles have the characteristics of spherical particles (size, 45–150 μm), irregular block particles (size, 30–70 μm) and irregular agglomerates (size, 30–50 μm), respectively.

**Figure 2:**
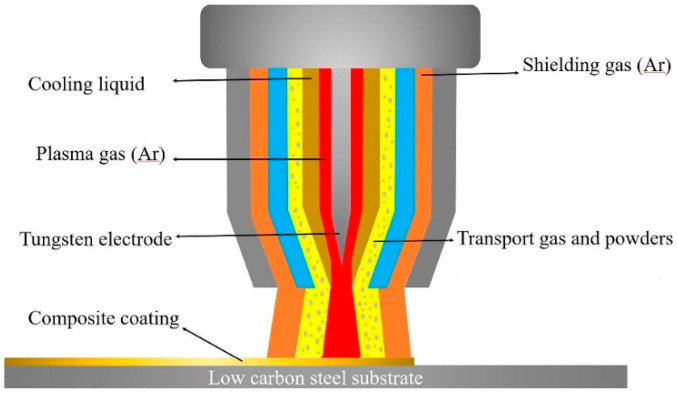
Schematic diagram of plasma spray welding.

NiCrBSi powders were selected so as to form the Ni matrix in the coatings, and Nb powders as well as NiCr-Cr_3_C_2_ powders were used to obtain the in-situ NbC particles. As shown in Table 2, in this investigation, P0, P1, P2, P3, P4, and P5 each represent the compositions of the mixed powders. In order to ensure the uniformity of the powders and prevent the powder from agglomerating, the powders were mixed with numerous steel balls in a powder vibrator for one hour. The welding equipment used in this study is the plasma spray welding machine (Shanghai Benxi Co., Ltd., Shanghai, China). Figure 2 illustrates the schematic diagram of plasma spray welding, and the selected parameters of the process are listed in Table 3.

Prior to testing, the samples for the analysis were cut, ground, and then polished. Afterwards, the polished samples were chemically etched by using the etchant of HNO_3_ and HF in a volume ratio of 10:1. The microstructure and elemental characterization of the composite coatings were investigated by scanning electron microscopy (SEM, SUPRA55, Carl Zeiss Co. LTD, Oberkochen, Germany) incorporated with an energy spectrum analyzer (EDS, Link-ISIS, Oxford Instruments Co. LTD, Abingdon, UK) and transmission electron microscopy (TEM, JEM-2100F, Japan Electronics Co. LTD, Tokyo, Japan). For the purpose of confirming the phases presented in the composite coatings, the Empyrean X-ray diffractometer (XRD, Malvern Panalytical Spectris Co. LTD, Almelo, Netherlands) with a working voltage and a current of 40 kV and 30 mA was used. The Cu target was used and the scanning speed was 4°/min.

To measure the micro-hardness along the depth of the composite coatings, a Vickers micro-hardness tester (HVA-50A, Laizhou Huayin Instruments Co. LTD, Laizhou, China) was employed under a load of 500 g for a duration of 10 s. The wear tests of the coatings were carried out by a wear testing machine (ML-100, Zhengli Machine Co. LTD, Foshan City, China). The samples with a dimension of 10 mm × 5 mm × 5 mm were fixed, and the wear tests were performed at a constant normal load of 19 N for 50 min. The grinding wheel was padded with the silicon carbon abrasive papers (360#). The wear tests were carried out on three samples for each coating. According to the average of the wear volume loss, the abrasive wear resistance was appraised. In addition, the worn surfaces were observed by SEM to investigate the wear mechanism.

## 3. Results and Discussion

### 3.1. XRD Analysis

**Figure 3:**
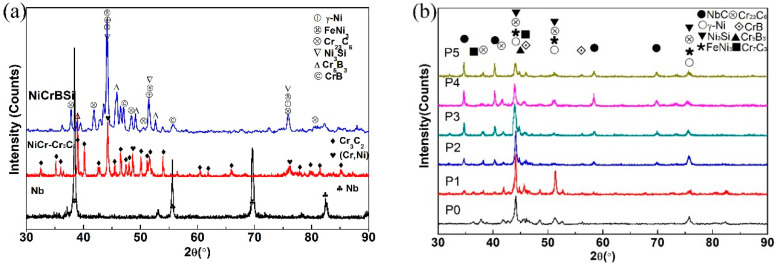
XRD patterns (**a**) powders and (**b**) coatings.

By use of X-ray diffraction (XRD), the phase compositions of the powders and coatings were analyzed, and the results are shown in Figure 3. On the basis of the diffraction peaks indexed results, in terms of JCPDS cards, there are γ-Ni, Cr_23_C_6_, Ni_3_Si, CrB, Cr_5_B_3_ and FeNi_3_ in NiCrBSi powders. NiCr-Cr_3_C_2_ powders mainly consist of Cr_3_C_2_ (and Ni, Cr). As shown in Figure 3b, it is found that γ-Ni, Cr_23_C_6_, Ni_3_Si, CrB, Cr_5_B_3_, Cr_7_C_3_ and FeNi_3_ occur in all coatings. In the composite coatings, no Cr_3_C_2_ phase presence can be found with powders P1–P5, which means the initial NiCr-Cr_3_C_2_ powders were almost completely decomposed. Besides, it is clear that the diffraction peaks of NbC are present in the composite coatings but not in the initial powders. That is to say, NbC particles were in-situ formed in the time of the process of the welding. The X-ray diffraction patterns of all the composite coatings are approximately identical, and the trends of the diffraction lines are also similar. However, the intensity of the NbC diffraction peaks, which are labeled as black dots, is stronger when the mass fraction of Nb and NiCr-Cr_3_C_2_ increases, and instead, the peak intensity of chromium carbides (Cr_23_C_6_ and Cr_7_C_3_) decreases relatively. This is because the synthesis of NbC needs some of carbons in the alloy solution and restricts the precipitations of chromium carbides. As a result, the increase in the mass fraction of Nb and NiCr-Cr_3_C_2_ will lead to the increase in in-situ NbC and the decrease in the quantity of chromium carbides in the composite coatings.

### 3.2. Microstructure

**Figure 4:**
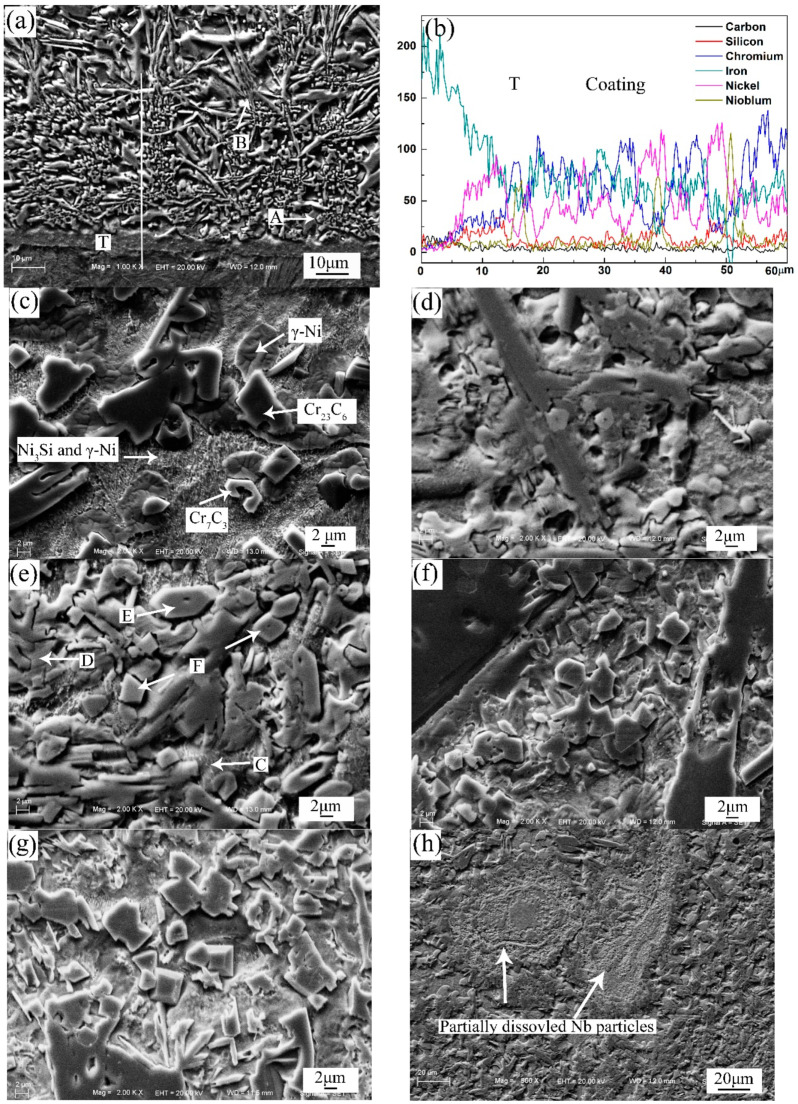
SEM microstructures of the coatings (**a**) coating with P1 near the fusion line, (**b**) energy spectrum analyzer (EDS) results, (**c**) coating with P0, (**d**) coating with P1, (**e**) coating with P2, (**f**) coating with P3, (**g**) coating with P4, and (**h**) coating with P5.

Figure 4 gives the typical microscopic structure of the coatings. Figure 4a shows the SEM microstructure of the coating with P1 near the fusion line. Obviously, there is a transition layer (T) with a width of 5–10 μm between the coating and the low carbon steel substrate. Meanwhile, no micro-pores or micro-cracks are observed in the coating. The EDS results along the line in Figure 4b show that there is a blend of the liquated powders and the steel substrate, which is proven by the fact that the transition layer is mainly composed by Ni and Fe elements. This further confirms that the composite coating is metallurgically bonded to the substrate. The microstructure of the coating bottom shows the character of rapid solidification. It is clear that there are many columnar crystals (A) with planar front growth perpendicular to the coating surface and a mass of dendrites (B). The EDS results for the marks are shown in Table 4. The results reveal that the columnar crystals (A) are relatively rich in Ni and Fe. Due to its high Ni concentration (62.99 at.% Ni), it should be a γ-Ni (Fe) solid solution. The dendrites (B) are determined as (Cr, Fe)_7_C_3_ based on the EDS results.

Figure 4c–h provides the SEM microstructure of the coatings with the designed powders. As shown in Figure 4c, the chromium carbide (Cr_23_C_6_ and Cr_7_C_3_) particles are distributed in the Ni matrix, and the Ni_3_Si, Cr_5_B_3_, CrB and FeNi_3_ particles are spread between the carbide particles in the coating with P0. This has been described in detail in our previous work [29]. The microstructure of the composite coatings with the addition of Nb and NiCr-Cr_3_C_2_ powders is different from that of the coating with P0. It can be observed from Figure 4e that the precipitates are distributed in the matrix (C) with different morphologies, such as lump phases (D), polygon phases (E) and blocky phases (F) with a uniform range of size. Since NiCrBSi powder is the main powder for the initial materials and the composition of the matrix (C) is 69.11 at.% Ni, the matrix should be a γ-Ni solid solution. The EDS results indicate that the chemical compositions of the lump phase (D) and the polygon phases (E) are 1.67 at.% Ni-3.75 at.% Fe-72.74 at.% Cr-17.49 at.% C and 1.36 at.% Ni-4.87 at.% Fe-55.26 at.% Cr-31.45 at.% C. According to the XRD results and Cr-C binary phase diagram, the lump phases are identified as Cr_23_C_6_, and the polygon phases are Cr_7_C_3_. The EDS results indicate that the blocky phases uniformly embedded in the matrix is enriched in Nb (39.37 at.%) and C (48.18 at.%), so they are identified as in-situ NbC. From Figure 4d–g, it is found that the increase in the mass fraction of Nb and NiCr-Cr_3_C_2_ can lead to an increase in NbC particles in the composite coatings, which is in accord with the XRD results shown in Figure 3. This can be put down to the increase in Nb and C concentrations in the molten pools. In addition, partially dissolved Nb particles can be seen in the composite coating with P5 (Figure 4h), because the plasma arc in this parameter was not enough to dissolve all the Nb powders completely. This will be harmful to the properties of the composite coatings.

**Figure 5:**
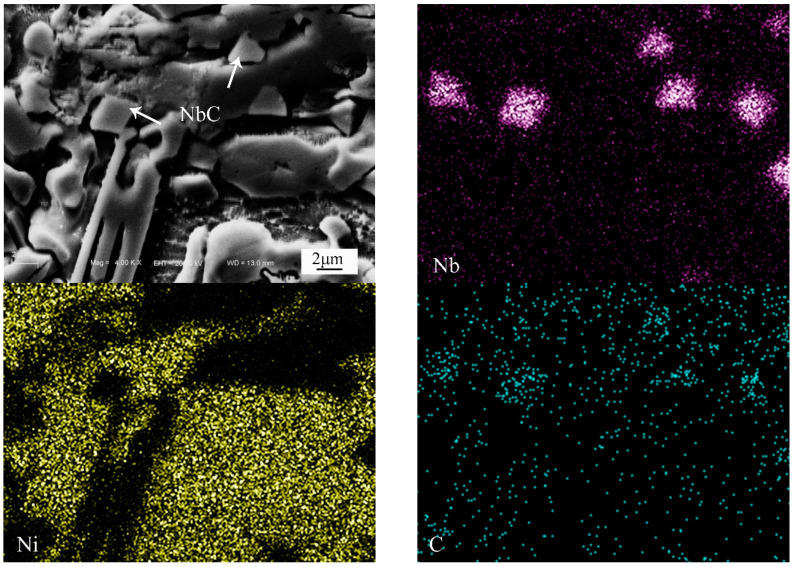

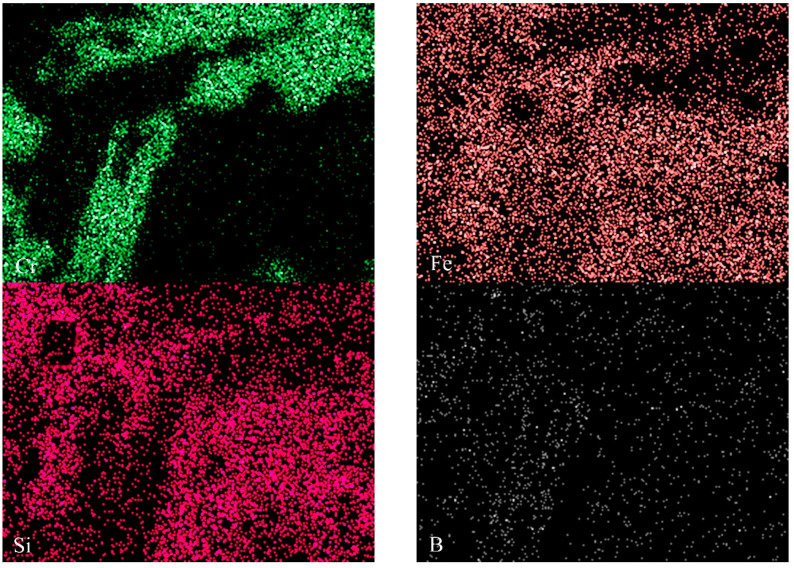
SEM microstructure and corresponding EDS analysis results of the composite coating with P4.

**Figure 6:**
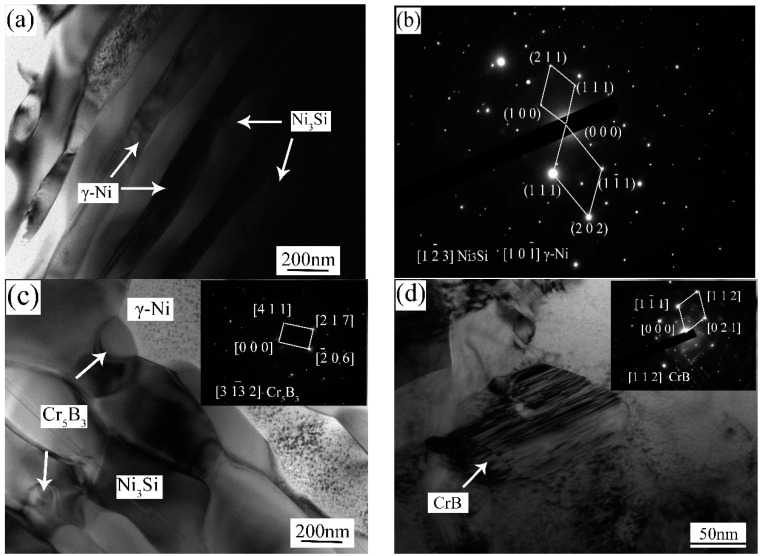

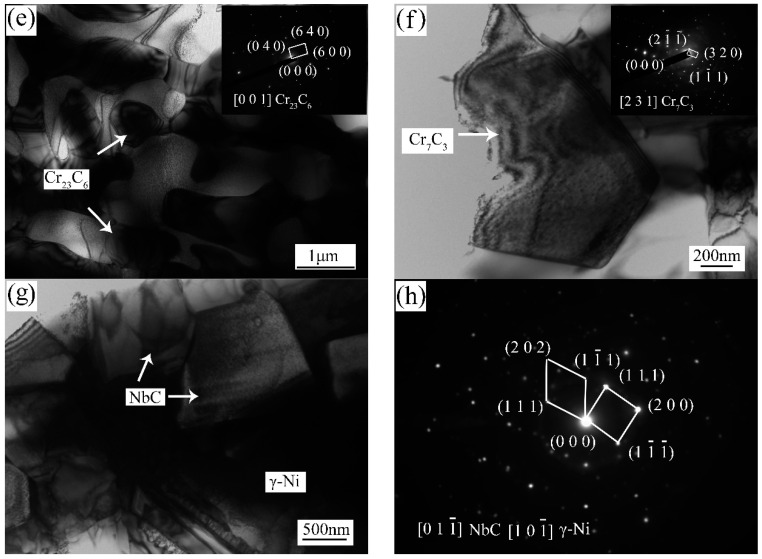
Transmission electron microscopy (TEM) images and selected area diffraction patterns (SADP) of coating with P4 (**a**) TEM image, (**b**) SADP of (**a**), (**c**) Cr_5_B_3_, (**d**) CrB, (**e**) Cr_23_C_6_, (**f**) Cr_7_C_3_, (**g**) NbC, (**h**) SADP of (**g**).

Figure 5 is the SEM microstructure and corresponding EDS analysis results of the composite coating with P4. As can be seen, Nb and C distribute evenly in the NbC particles, and Cr shows no signs of enrichment. Fe, Si and B distribute between the particles and the matrix. Hence, the Ni_3_Si, Cr_5_B_3_, CrB and FeNi_3_ phases mainly spread in this region in the composite coating. TEM was used to further reveal the microstructural details of composite coatings. Figure 6 shows the bright filed TEM images and selected area diffraction patterns (SADP) in the composite coating with P4. By calculating the selected area diffraction pattern (Figure 6b), alternating laminas of Ni_3_Si and γ-Ni phases can be observed; see Figure 6a. According to the chemical compositions (EDS: 70.13 at.% Ni, 6.31 at.% Fe, 3.62 at.% Cr, 17.45 at.% Si, 2.16 at.% C and 0.33 at.% B), it is confirmed that the white phase is Ni_3_Si. It is also clear that Cr_5_B_3_, CrB, Cr_23_C_6_ and Cr_7_C_3_ particles in nanometer sizes exist in the composite coating. As depicted in Figure 6g, it can be ascertained that the particle is NbC by the SADP. It is clear that in-situ NbC with a face-centered cubic structure is in the sizes of 0.5–2 μm. In addition, the TEM image also reveals that there are no deleterious phases between the in-situ NbC particle and γ-Ni matrix, and the interface is clean. Hence, there is a strong bonding between the in-situ NbC particles and the matrix, and it is favorable to the improvement of the properties.

NiCrBSi powders and the steel substrate first melt because of the lower melting point and then form a molten pool with Ni-alloy solution in the time of the plasma spray welding. In the molten pool, the concentrative plasma arc with high temperature can promote the decomposition of Cr_3_C_2,_ resulting in raising the concentrations of Cr and C, and there is enough time for a reaction of NbC between Nb and C. Since NbC has the higher melting point and the more negative free energy of formation compared with other chromium carbides, NbC particles separate out from the molten pool once they are formed. Then, the chromium carbides are formed and separate out from the liquid phase due to the raising solute concentrations. As the solidification proceeds, chromium borides, Ni_3_Si and γ-Ni gradually form around NbC particles [29]. In conclusion, in-situ NbC and chromium carbide are the reinforcement particles in the Ni matrix, and hence they can be called in-situ NbC reinforced composite coatings.

### 3.3. Micro-Hardness

**Figure 7:**
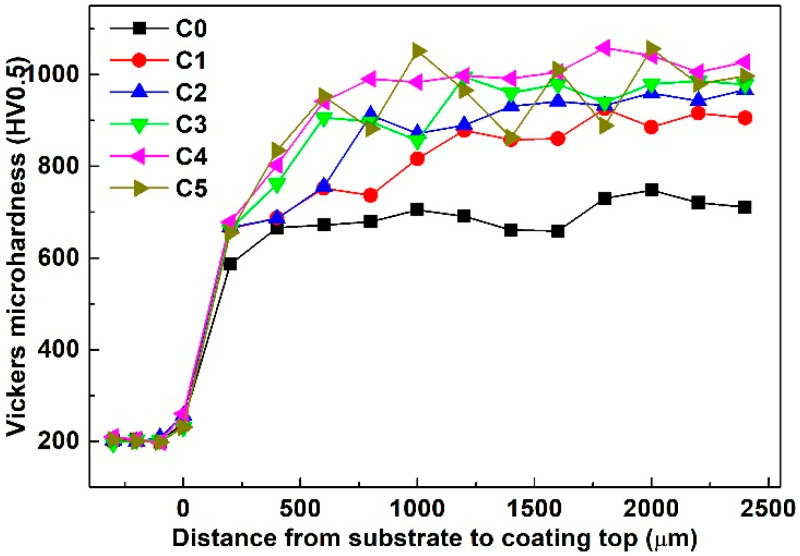
Micro-hardness distribution curves perpendicular to the direction of the fusion line.

To express the coatings conveniently in this study, C0, C1, C2, C3, C4 and C5 are used to represent the composite coating prepared by the powder which were numbered as P0, P1, P2, P3, P4 and P5. The micro-hardness distribution curves perpendicular to the direction of the fusion line are shown in Figure 7. The micro-hardness of all the coatings is prominently higher compared with that of the low carbon steel, and the micro-hardness of C0 is lower than that of the in-situ NbC reinforced composite coatings. The average micro-hardness of C4 reaches the maximum value 1025HV_0.5_, which is about 5.1 times that of the substrate (201 HV_0.5_) and 1.5 times that of C0 (682 HV_0.5_). Moreover, an increase in the mass fractions of Nb and NiCr-Cr_3_C_2_ in the raw materials will lead to an increase in the micro-hardness of the composite coatings. It is well known that NbC particles have high hardness, and the chromium carbide particles also have high hardness [29]. Numerous in-situ NbC particles and chromium carbide particles spread over the matrix in the composite coatings. Therefore, the micro-hardness of the composite coatings is improved significantly. Besides, dislocation motion will be impeded, resulting from the existence of the weeny precipitates such as CrB, Cr_5_B_3_, FeNi_3_ and Ni_3_Si. The micro-hardness of the composite coatings increases with the increase in the mass fractions of Nb and NiCr-Cr_3_C_2_ powders, which can be attributed to the increased content of in-situ NbC particles. Obviously, the variation tendency of the micro-hardness of C5 is not smooth, and the micro-hardness fluctuates up and down. This can be attributed to the presence of partially dissolved Nb particles.

### 3.4. Wear Resistance

**Figure 8:**
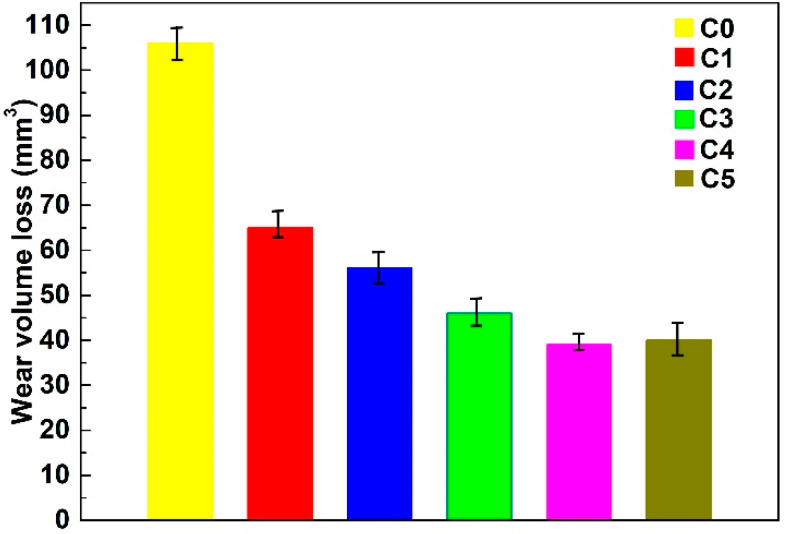
Wear volume loss of the composite coatings.

In this study, the wear volume loss was used to evaluate the wear resistance. The less the wear volume loss, the better the wear resistance is. The volume loss of the substrate and the coatings is presented in Figure 8. As indicated, the volume losses of C0, C1, C2, C3, C4 and C5 are 106.1 mm^3^, 65.2 mm^3^, 56.7 mm^3^, 46.9 mm^3^, 39.2 mm^3^, and 40.1 mm^3^, yet that of the substrate is up to 625 mm^3^. It is apparent that the composite coatings exhibit superior wear resistance. In addition, it is clear that the volume loss of the composite coatings decreases as the content of Nb and NiCr-Cr_3_C_2_ powders increases. Especially, composite coating C4 gives the least wear volume loss, and its volume loss is only 37% of that of C0 and 6% of that of the substrate under the identical conditions in this study. However, there is a small increase in the volume loss of C5. The reason could be attributed to the non-uniform micro-hardness resulting from the partially dissolved Nb particles.

**Figure 9:**
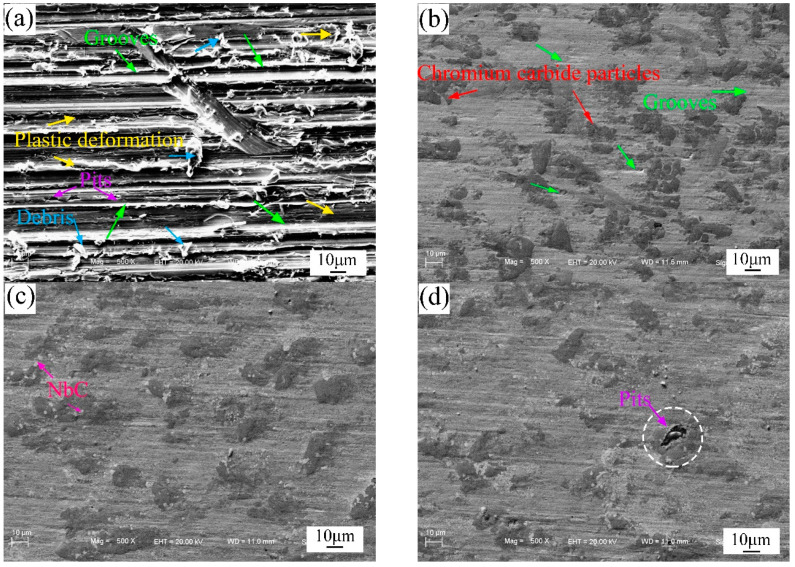
Worn surface morphologies of the substrate and the composite coatings (**a**) substrate, (**b**) C0, (**c**) C4, and (**d**) C5.

SEM images of worn surfaces of the substrate and the composite coatings are shown in Figure 9, which can help to comprehend the wear mechanism of in-situ NbC reinforced NiCrBSi composite coatings. As illustrated in Figure 9a, the worn surface of substrate has typical abrasive and adhesive wear features evidenced by numerous detaching debris, deep ploughing grooves and serious plastic deformation characteristics. The hard abrasive particles can plough and cut the substrate without a hitch. Though C0 has relatively slight grooves, there still exists evident grooves in the soft matrix. This demonstrates that the chromium carbide could retard the cutting of the abrasive particles, whereas the comparatively soft matrix could not. It is noted that the width and depth of the wear scar of the composite coatings are much smaller compared with C0. This confirms that the composite coatings have a more prominent wear resistance than C0, which is also consistent with the wear volume loss results. However, some pits are detected in C5 (Figure 9d). As demonstrated by the results of the SEM analysis, some partially dissolved Nb particles are visible in coating C5 (Figure 4f). Thus, the presence of pits can be ascribed to partially dissolved Nb particles being ground off.

The wear mechanism of in-situ NbC reinforced composite coatings is dependent on several factors such as material hardness, microstructural features and wear conditions. As noted previously, a number of fine in-situ NbC particles and numerous chromium carbide particles are distributed in the γ-Ni matrix in the composite coatings. Due to the high hardness of NbC and chromium carbides, the abrasive grains can only act on the relatively soft γ-Ni matrix. When the abrasive grains meet the NbC reinforcements, the reinforcements play an important role in resisting the grinding and squeezing of the abrasive grains. Obviously, the wear scars observed on the worn surface of the composite coatings are discontinuous. During the wear tests, γ-Ni matrix constantly undergoes micro-cutting resulting in the exposure of the NbC particles to the surface of composite coatings, and hence the NbC particles could prevent the abrasive grains more effectively. Since the NbC particles with face-centered cubic structures are in-situ synthesized and could be wet by γ-Ni matrix with the same crystal structure, the bonding strength between NbC particles and Ni matrix is improved. Moreover, the references have proved that the NiCrBSi coatings have a good toughness [30,31]. The Ni matrix can support the particles and protect them from felling off. So, the micro-cutting is the main wear mechanism of in-situ NbC reinforced composite coatings. However, the excessive addition of Nb and NiCr-Cr_3_C_2_ powders could not improve the wear resistance. On the contrary, the wear resistance of C5 is decreased due to the partially dissolved Nb particles which have a poor bond with the matrix and are prone to fall off.

## 4. Conclusions

(1) In-situ NbC reinforced Ni-based composite coatings were successfully fabricated by plasma spray welding with the powder mixtures of NiCrBSi + Nb + NiCr-Cr_3_C_2_. XRD results showed that the main phase of the composite coatings were NbC, γ-Ni, Cr_23_C_6_, Ni_3_Si, CrB, Cr_5_B_3_, Cr_7_C_3_ and FeNi_3_. In-situ NbC with a uniform size and chromium carbide were the reinforcement particles in the composite coatings. The content of NbC particles increased with the increase in the mass fractions of Nb and NiCr-Cr_3_C_2_ in the raw materials.

(2) On account of the presence of in-situ NbC particles and the chromium carbide particles, the micro-hardness and the wear resistance of the composite coatings were significantly improved compared with that of the NiCrBSi coating. The wear mechanism of the composite coating is micro-cutting. The wear resistance of the composite coating with the powder mixtures of 20% (Nb + NiCr-Cr_3_C_2_) and 80% NiCrBSi is the best in this study.

## Figures and Tables

**Table 1:** The chemical compositions of the raw materials (wt.%).

Powders	Cr	Fe	Si	B	C	Ni
NiCrBSi	17	8.0	4.0	3.5	1.0	Bal.
NiCr-Cr_3_C_2_	Bal.	0.3	0.4	-	9.0	24.4

**Table 2:** The compositions of the powders used in this research (wt.%).

Mixed Powders	P0	P1	P2	P3	P4	P5
NiCrBSi	100	95	90	85	80	75
Nb	-	2.82	5.64	8.46	11.28	14.10
NiCr-Cr_3_C_2_	-	2.18	4.36	6.54	8.72	10.90

**Table 3:** Welding parameters.

Welding Current (A)	Plasma Gas (L/min)	Transport Gas (L/min)	Shielding Gas (L/min)	Welding Speed (mm/s)	Welding Distance (mm)	Powders Feeding Speed (g/min)	Nozzle Diameter (mm)
128	2.1	6.3	13	0.9	12	9	2.4

**Table 4:** EDS results.

Marked Locations	Elements (at.%)							Phase
	Ni	Nb	Cr	C	Si	B	Fe	
A	62.99	1.01	3.71	2.68	3.16	0.76	25.69	γ-Ni (Fe)
B	7.68	5.37	36.15	28.98	1.62	1.95	18.25	(Cr, Fe)_7_C_3_
C	69.11	7.36	2.23	0.43	10.83	2.94	7.10	γ-Ni
D	1.67	2.05	72.74	17.49	1.18	1.12	3.75	Cr_23_C_6_
E	1.36	3.44	55.26	31.45	2.76	0.86	4.87	Cr_7_C_3_
F	3.42	39.37	4.85	48.18	0.25	3.07	0.86	NbC
